# Cross-cultural adaptation and initial validation of the Brazilian-Portuguese version of the pediatric automated neuropsychological assessment metrics

**DOI:** 10.3389/fpsyg.2022.945425

**Published:** 2022-09-16

**Authors:** Jaqueline Cristina de Amorim, Simone Thiemi Kishimoto, Cibele Longobardi Cutinhola Elorza, Flávia Alegretti Cavaletti, Roberto Marini, Clovis Artur Silva, Claudia Saad-Magalhães, Paula Teixeira Fernandes, Hermine I. Brunner, Simone Appenzeller

**Affiliations:** ^1^Post-graduate Program of Child and Adolescent Health, School of Medical Science, University of Campinas, Campinas, Brazil; ^2^Laboratory of Autoimmune Diseases, School of Medical Science, University of Campinas, Campinas, Brazil; ^3^Department of Pediatrics, School of Medical Science, University of Campinas, Campinas, Brazil; ^4^Child and Adolescent Institute, Clinical Hospital (HCFMUSP), São Paulo of University, São Paulo, Brazil; ^5^Pediatric Rheumatology Division, Botucatu School of Medicine, São Paulo State University (UNESP), São Paulo, Brazil; ^6^Department of Sport Sciences, Faculty of Physical Education, University of Campinas, Campinas, Brazil; ^7^Division of Rheumatology, Children’s Hospital Medical Center, University of Cincinnati, Cincinnati, OH, United States; ^8^Department of Orthopedics, Rheumatology, and Traumatology, School of Medical Science, University of Campinas, Campinas, Brazil

**Keywords:** Ped-ANAM, cognition, translation, validation, psychology, Brazilian-Portuguese

## Abstract

Automated neuropsychiatric batteries have been used in research and clinical practice, including for chronic diseases, such as Systemic Lupus Erythematosus. The Pediatric Automated Neuropsychological Assessment Metrics battery (Ped-ANAM), originally developed for use in American-English speaking individuals, allows tracking of cognitive functions. It can be applied to people over 9 years old. The aim of this study was to translate and present initial validation data from the Ped-ANAM into Brazilian-Portuguese. We translated the battery according to Beaton’s guidelines. Psychometric properties were tested, internal consistency was analyzed by Cronbach’s alpha coefficient, test-retest reliability by the intraclass correlation coefficient (ICC). Further, we measured the test execution speed at both times as a temporal stability. Principal component analysis (PCA) was used for structural validity. Evidence of construct validity was assessed through assessment of the relationships with the Wechsler Intelligence Scales. All participants prior to the start of study related activities signed an informed consent form approved by the local ethics committee. A sample of 230 individuals [mean (range) of age: 23 (9 to 60) years; 65% females] was included; a subset of 51 individuals [mean (range) of age: 18 (9 to 57) years, 59% female] completed the Ped-ANAM twice to assess test-retest reliability, and another subset of 54 individuals [mean (range) of age: 20.4 (7 to 62) years; 67% female] completed the Wechsler Intelligence Scales for Children and Adult for assessment of the Ped-ANAM’s construct validity. Our results suggest that the internal consistency of the Ped-ANAM (Cronbach’s α = 0.890) and its subtest test-retest reliability were excellent (ICC: 0.59 to 0.94). There was no clustering in the Principal Components Analysis, suggestive of non-grouping of the evaluated variables. Construct validity assessment to the Wechsler Scales showed expected ranges of low to strong correlations (Spearman correlations: ρ = 0.40 to ρ = 0.69). We concluded that, based on the results of this study, a cross-culturally validated Brazilian-Portuguese version of the Ped-ANAM has been developed and it is a reliable tool for the screening cognitive function.

## Introduction

Cognition is a psychological function responsible for the acquisition of knowledge; therefore, it is extremely important for the structuring of childhood development and can be considered a performance indicator (Bordin et al., 2001). According to the Diagnostic and Statistical Manual of Mental Disorders (DSM) (American Psychiatric Association, 2013) there is a difference between intellectual disability which is defined as a neurodevelopmental disorder and Cognitive Impairment (CI), characterized by the loss of initially healthy cognitive functioning, and is considered present when cognitive performance is lower than expected for age and schooling. CI also needs to be distinguished from the diagnosis of dementia that commonly impairs activities of daily living (Bordin et al., 2001; Petersen and O’Brien, 2006; American Psychiatric Association, 2013).

Some medical conditions can cause CI, such as: primary or secondary brain tumors, subdural hematoma, endocrine conditions (e.g., hypothyroidism, hypercalcemia, hypoglycemia), nutritional conditions (e.g., thiamine or niacin deficiencies), other infectious diseases, and, finally, immunological disorders (e.g., temporal arteritis, systemic lupus erythematosus). CI may also occur concurrently with intellectual disability (e.g., a person with Down Syndrome who develops Alzheimer’s disease or a person with an intellectual disability who loses slightly more cognitive ability after a traumatic brain injury) (American Psychiatric Association, 2013). Regarding CI in chronic diseases, The American College of Rheumatology (ACR), *Ad Hoc* Committee on Neuropsychiatric Lupus Nomenclature, operationally defined CI as significant deficits in any or all of the following major cognitive functions: complex attention, executive skills (e.g., planning, organizing, sequencing), memory, learning, recall, visual-spatial processing, language, and psychomotor speed. Such demands can be subtle and fluctuate over time (Liang et al., 1999; Petersen and O’Brien, 2006; Yuen et al., 2021). In this study, our objective is to measure IC.

The gold standard criterion for the diagnosis of CI is standardized neuropsychological tests performed by mental health professionals (Petersen and Morris, 2004). There are complete tests and combinations of subtests for each specific area. The various severity levels are defined based on adaptive functioning, not intelligence quotient scores, as it is adaptive functioning that determines the level of support needed (American Psychiatric Association, 2013). Unfortunately, formal neuropsychological testing is expensive, time-consuming, and not easily accessible during daily clinical practice (Brunner et al., 2007). In this context, computerized batteries have become important CI assessment and screening tools. Also, because they offer a promising approach, they have gained popularity over the last 10 years (Kane and Kay, 1992; Gottschalk et al., 2000; Kabat et al., 2001; Yuen et al., 2021).

Among these, there is the ANAM (Automated Neuropsychological Assessment Metrics) Test System, which is a comprehensive set of computer-based tools for use in assessing cognition. The system includes tools for testing, reporting, and data management. ANAM software is an automated and self-administered tool and performs the screening of changes in cognition, a quick tool to administer (30–40 min) (Reeves et al., 1996; C-Shop, 2007; Robert et al., 2007; Yuen et al., 2021).

It was originally developed, in English, by the Office of Military Performance Assessment Technology, designed for clinical and research applications (Robert et al., 2007). It is used in the military area, which includes research done using the ANAM as a key tool in assessing the cognition of military personnel and veterans (Reeves et al., 1996; Gottschalk et al., 2000; Gamache et al., 2005).

There are also studies carried out using the ANAM in a sports context. Much, though not all, of this research is devoted to ANAM in concussion management (Reeves et al., 1996; Cernich et al., 2007). Finally, its use is mainly made in general health research, examples include CI screening in patients with chronic diseases, such as: Systemic Lupus Erythematosus, Fibromyalgia, Multiple Sclerosis, cognitive effects of substance abuse (Kane and Kay, 1992; Gottschalk et al., 2000; Kabat et al., 2001; Robert et al., 2007), result of radiation exposure (Gamache et al., 2005), Alzheimer’s disease and Parkinson’s disease (Levinson et al., 2005). ANAM should be administered by professionals with trained, recommended ANAM administration procedures, following the general guidelines of the American Psychological Association (APA) (American Psychological, and Association, 2000; American Educational Research Association [AERA] et al., 2014) for the distribution and administration of psychological tests (Reeves et al., 1996; Reeves and Bleiberg, 2004).

There are different individual tests in the ANAM test library (Kane and Kay, 1992). These individual modules can be grouped into different test batteries. Commonly used batteries include the ANAM GNS battery ANAM Core battery (Meyers et al., 2022) and, more and more recently, a pediatric version, the pediatric automated neuropsychological assessment metrics (Ped-ANAM), which has been used to assess cognitive ability in chronic diseases (Shrout and Fleiss, 1979; Cernich et al., 2007; Meyers et al., 2022).

It is an adapted version of the ANAM software with nine subtests. The main difference is the more playful language that, before, could only be understood by people over 13 years old, but now, has undergone a change to be understood by children from 9 years old. An important advantage of this change is that it is mostly graphical, many of the tasks of Ped-ANAM are almost identical to traditional ANAM so the device can be administered to children, teenagers, and adults (Kane and Kay, 1992; Reeves et al., 1996; Reeves and Bleiberg, 2004; Brunner et al., 2007). As it is applicable throughout a patient’s lifetime, Ped-ANAM seems especially suited for monitoring cognitive ability over time. Thus, combining greater practicality in administration, Ped-ANAM can be used to test cohorts that cover a wide range of ages, or cohorts, with participants that cross traditional-age categories, for example, childhood, adolescence, and adulthood. Administration of Ped-ANAM requires 30–40 min, as does ANAM (Kane and Kay, 1992; Reeves et al., 1996; Reeves and Bleiberg, 2004; Brunner et al., 2007).

The Ped-ANAM battery used in this study requires a Microsoft Windows operating system and a USB-connected mouse (Reeves et al., 1996; Reeves and Bleiberg, 2004; Brunner et al., 2007). It is a user-friendly, easy, and intuitive program, supporting its acceptability and ease of use in clinical practice. There is a fixed format, the sequence of activities occurs in the same way in all applications. Before performing each battery subtest, there is an ambience system to understand the activity. At this moment, the participant responds, and hits and misses are not counted. Positive signs are shown on the screen, after hits and negative signs, in case of mistakes. In case of excessive error rates, there is a brief interruption. The objective is to train the activity and confirm the interviewee’s understanding (Reeves et al., 1996; Reeves and Bleiberg, 2004).

The Ped-ANAM battery has been used in the area of rheumatology, both in clinical and research environment, showing promise due to the practicality of its application and also the ability of the software to identify CI at different moments in time (Reeves et al., 1996; Reeves and Bleiberg, 2004). In addition, the Ped-ANAM is a screening tool that can help identify patients with CI and optimize the use of public health resources in a country with limited economic resources. After the Ped-Anam has been translated into Brazilian Portuguese, it will be possible to use it in research with patients with Systemic Lupus Erythematosus, multicentric and longitudinal research, as the battery is different because it is faster and less expensive in practice compared to others currently used (AlE’ed et al., 2017). The requirements for use after the translation process will be the same as those already used in the original version of the battery in English, age group from 9 years old, calm environment for application, computer and mouse properly installed. Thus, the objective was to carry out the translation, cross-cultural adaptation and, later, to present the reliability and evidence of the validity of the Ped-ANAM in Brazilian Portuguese.

## Materials and methods

### Study design

A quantitative, observational, descriptive cross-sectional study was conducted in the countryside of the state of São Paulo, Southeastern of Brazil, to assess the measurement properties of the translated version of Ped-ANAM for use in this country.

### Participants

The study was carried out in Campinas, Brazil. The convenience sample consisted of 230 participants following the psychometric theory of Nunnally (1978), which recommends a minimum of 10 items for each item of a scale, so that Ped-ANAM would have 9 Subtests that could be validated. Also corroborating with findings by Tabachnick and Fidell (2007). Participants who met the study criteria were asked to complete a test in a quiet room and were accompanied by a trained investigator. All of them were 9 years old or older, the mean age was 23 (± 11 years; range = 9 to 60 years). Volunteers with a history of CI were excluded. From this sample, a group of 51 [mean age 18 years; range = 9 to 57 years; (58.82% female)] people were invited and answered the test for the second time, and other 54 volunteers [mean age 20.4 years; range = 9 to 62 years; 36 were (66.67% female)] were invited to answer as Wechsler Intelligence Scales. Seventeen (31.48%) took a Wechsler Intelligence Scales for Children (WISC) and 37 (68.52%) responded to the Wechsler Adult Intelligence Scale (WAIS).

### Data collection procedures

The project was approved by the local Ethics Committee (CAAE 39750914.3.1001.5404). All volunteers were personally contacted, informed about the study, invited to participate, and voluntarily signed an informed consent form. A researcher trained psychologist applied the Wechsler Intelligence Scales to the volunteers, and the average time for this was 105 min (range 100–110 min). All participants responded to the test in an individualized environment, without interruptions.

#### Instruments

**FIGURE 1:**
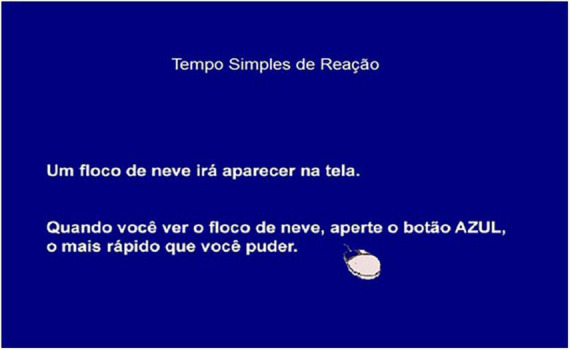
Ped-ANAM screen in the simple reaction time subtest 1.

The Ped-ANAM is composed of 9 subtests that are always performed in the same order (Table 1). For their application it is necessary to have a computer and a mouse, as the cognitive tasks are read on the screen (Figure 1) and answered using the cursor, which contains two colors: Red or blue (Reeves et al., 1996; Reeves and Bleiberg, 2004). In addition, the Ped-ANAM test has an initial feature where the software performs an initial test that checks the participant’s understanding before performing the main activity (Bauer et al., 2012).

**TABLE 1:** Ped-ANAM subtests and their functions in order of execution.

Test	Domain/Function	Description of tasks*
Sleepiness scale	Sleepiness	The user is presented with seven different faces representing a range of alertness/sleepiness, ranging from “Feeling very alert, wide awake, and energetic” to “Very sleepy and cannot stay awake much longer.”
Simple reaction time	Basic neural processing (Emphasis on motor activity)	A series of “*” symbols is presented on the display. The user is instructed to respond as quickly as possible by pressing a button each time the stimulus appears.
Procedural reaction time	Information processing speed, visuomotor reaction time, simple decision making, and attention.	The user is presented with a number (either a 2, 3, 4, or 5). The user is instructed to press one designated button for a “low” number (2 or 3) and another designated button for a “high” number (4 or 5).
Code substitution—learning	Visual scanning, visual perception, attention, associative learning, and information processing speed.	The user must compare a displayed digit-symbol pair with a set of defined digit-symbol pairs presented at the top of the screen. The user presses designated buttons to indicate whether the pair in question is correct or incorrect relative to the key.
Logical relations	Reasoning and verbal syntax	The user must decide if sentences presented on the screen make sense or not. The user presses designated buttons to indicate whether the sentence makes sense or not.
Spatial processing	Spatial processing	Two four-bar histograms are presented, the first of which is displayed upright and the second of which is displayed after a 90degree rotation, either clockwise or counter-clockwise. The user presses designated buttons to indicate if the two histograms are the same or different, regardless of the orientation.
Running memory continuous performance test	Working memory	Single characters are presented on the display in rapid sequence. The user presses designated buttons to indicate if the displayed character matches or does not match the preceding character.
Mathematical processing	Assesses basic computational skills, concentration, and working memory	An arithmetic problem is presented on the screen and the user must decide if the problem is correct or incorrect. Each problem involves one mathematical operation (addition or subtraction) on two single-digit numbers. The user presses buttons to indicate whether the presented problem is correct or incorrect.
Matching grids	Spatial Processing	During this test, the user must determine if two grid patterns are identical except for a possible rotation. The two grid patterns are presented, side by side, on the display. The user presses designated buttons to indicate whether the grid patterns match or do not match.
Matching to sample	Visual-spatial processing, working memory, and visual short-term recognition memory.	A patterned grid appears and disappears for five seconds, then, two grids are displayed, side by side. One of these grids matches the previous grid and the examinee presses the button corresponding to the correct match side.
Memory search	Short term memory	A set of six characters is displayed for memorization. Individual characters are then displayed and the user presses designated buttons to indicate if each character is a member of the memorized set or not.
Code substitution—delayed	Learning and delayed visual recognition memory	Is presented with a digit-symbol pair and must decide from memory if this pairing is correct based on the key presented during the Code Substitution – Learning test taken earlier in the test battery.

*Adapted from Reeves and Bleiberg (2004) and Cognitive Science Research Center [Csrc] (2014).

After the application is finished, the battery data is saved at system completion for each of the 9 individual subtests and its scores are automatically generated by this data collection system. Both main measurements and runtime relationship measurements in the subtests are generated in more detail (Table 2):

**TABLE 2:** Descriptions of variables automatically generated by Ped-ANAM battery*.

Correct	Correct number of trials with a correct response
Incorrect	Incorrect number of trials with an incorrect response
Lapse	Lapse number of trials where no response was made in the allotted time
Speed	The speed at which the subtest was answered
Mean response time (in milliseconds) score:	Average response time for correct responses (milliseconds)
Percentage correct Score:	Percent of items with a correct response
Throughput Score:	Number of correct responses per minute

*Adapted from Reeves and Bleiberg (2004).

All ANAM batteries have the same standard scoring system, and the Throughput Score is considered a measure of cognitive effectiveness or efficiency and is a combination of reaction time and accuracy (Reeves et al., 1996; Reeves and Bleiberg, 2004). In addition to individual scores for each subtest, the battery also features a metric called “effort,” which can range from 0 to 33; the lower the score, the better the result. The cut-off point is 14, with 0–14 being good performance and 15–33 not being a good effort, an indication of CI (Reeves et al., 1996; Reeves and Bleiberg, 2004). Recently, with the increased use of Ped-ANAM battery in healthcare, a lupus IC research group developed four candidates for performance indices called the cognitive performance score (CPS) (Vega-Fernandez et al., 2015). It is a combination of different subtest scores, and this data subset underwent different statistical approaches, such as: Unweighted averages of accuracy scores of all Ped-ANAM subtests; accuracy scores of all Ped-ANAM subtests weighted through principal component analysis (PCA); algorithm derived from logistic models.

### Procedure

Authorized by its owner, Ped-ANAM was translated into Brazilian-Portuguese and adapted according to the method proposed by Beaton et al. (2000):

Step 1: The initial battery version, prepared in English language, was translated into Brazilian-Portuguese by two bilingual translators with Brazilian-Portuguese language as their mother tongue, performing the translations independently. One of the translators knew the basic objectives of the tool and was an area specialist, while the other was not connected to the area. The translators were instructed to use simple language (Beaton et al., 2000).

Step 2: The translated versions were evaluated by a bilingual expert to verify the translation, and it was considered whether each item kept the same meaning for the culture of interest. The consensus version was elaborated giving importance to semantic similarity (Beaton et al., 2000).

Step 3: Two other native Brazilian translators were responsible for performing a back-translation into English. None of the back-translators knew the topic addressed by the evaluation tool an important requirement to know if the version corresponded to the original content (Beaton et al., 2000).

Step 4: A committee of experts was assembled, and all the notes previously made were taken to the committee along with all the material produced; content evaluation was performed as described in Table 3. From then on, the production of a pre-test version started, which should be understood by a child over 9 years old and be equivalent to the original instrument in four aspects: semantic, experimental, idiomatic, and conceptual (see Table 3). Then, at the time of analysis for comprehension, a group of volunteers, composed of five people representing each age group, answered the questionnaire about battery compression. Words with a degree of difficulty were replaced by colloquial words for everyday use to give more understanding to the original expression, as described in Supplementary File 1. The committee could modify or reject the format and items, as well as add new ones. The final version of Ped-ANAM in the Brazilian-Portuguese language was submitted to the authors of the protocol, who were aware of all the steps previously performed. Then, the protocol was approved for use after the review (Beaton et al., 2000).

**TABLE 3:** Stage IV of translation and adaptation: Equivalences*.

Semantics	Grammar and vocabulary analysis.
Idiomatic	Colloquial expressions can be difficult to translate
Experimental	Terms used must be consistent with the routine and experience of the population of interest. Even if it is translatable, certain tasks may not be seen in different countries.
Conceptual	Words often have different concepts and meanings across cultures

*Adapted Beaton et al. (2000).

Step 5: The pre-test version, established by the specialists committee, was applied in a group of volunteers of age groups that contemplate the age of application of the battery (children, teenagers, adults, and elderly) to verify the comprehension of the items. The volunteers who participated in the study contributed to the pre-test version of the battery to assess clarity, understanding and application. If there were doubts or difficulties regarding the application, the professional could propose phrases or terms that should be more understandable and compatible with reality. The suggestions made by the volunteers during the pre-test were taken to the expert committee, where the final version in Brazilian-Portuguese was defined (Beaton et al., 2000).

Step 6: The final stage of the adaptation process was the submission of all reports and forms to the developer and to the committee that accompanied the translated version to check the recommended steps (Beaton et al., 2000).

#### Psychometric properties

After the translation process, we analyzed the reliability and validity of the battery. The techniques were in accordance with The Standards for Educational and Psychological Testing (Bauer et al., 2012; American Educational Research Association [AERA] et al., 2014).

#### Reliability

Internal consistency: After translation, the battery was applied to our sample. The Ped-ANAM was completed without problems by all enrolled subjects. Questions were well understood. The median time complete the Ped-ANAM battery was 40 min (range: 35–45 min).

Test-retest reliability: It was evaluated, through the application, in 51 volunteers who responded to the battery twice with an interval of 10 days between applications, and the guidelines and evaluation conditions were the same on both occasions. A trained researcher applied the battery in volunteers of both sexes. To analyze the results of these applications, we analyzed the performance against errors and successes and also the speed of responses, thus evaluating the variation of time in two moments (Bauer et al., 2012; American Educational Research Association [AERA] et al., 2014).

#### Validity

##### Evidence based on internal structure

Internal structure analysis: Based on the results of the main sample, we analyzed the results of 230 participants using the number of correct answers in each subtest as input items.

##### Evidence based on relations to other variables

Convergent Validity: For the analysis of construct validity, a group of 54 volunteers responded to the Ped-ANAM and the Wechsler Intelligence Scales, a battery already accepted and valid, considered the gold standard (Hartman, 2009). The application was performed by an experienced researcher, a psychologist, and the Wechsler batteries were divided according to age group. The WISC is an instrument for children and teenagers, aged 6–16 years and 11 months, consisting of 10 main and 5 complementary subtests, including: verbal comprehension, abstract reasoning, perceptual organization, quantitative reasoning, memory and speed process. For this age group, we have a group of 16 volunteers of both sexes (Zanarella, 2015). The WAIS, on the other hand, is an instrument for teenagers and adults, aged between 16 and 89 years, composed of 15 subtests that include verbal comprehension, organization perception, processing speed and working memory. For this age group, we have 37 volunteers (Cronbach, 1951; Wechsler, 1997).

### Data analysis

Statistical analyses were performed using The SAS System for Windows (Statistical Analysis System), version 9.4 SAS Institute Inc., 2002–2012, Cary, NC, United States, and The SPSS System for Windows (Statistical Product and Service Solutions), version 20, IBM.

#### Reliability

For the analysis of internal consistency reliability, Cronbach’s alpha coefficient was used, drawing on the following classification: α 0.9 0.9: excellent; 0.9 > α ≥ 0.8 good; 0.8 > α ≥ 0.7: acceptable; 0.7 > α ≥ 0.6 questionable; 0.6 > α ≥ 0.5 bad; and 0.5 < α unacceptable (Cronbach, 1951). The utilized Ped-ANAM scores were the number of correct answers for each subtest.

To assess the test/retest reliability of the instrument, regarding its stability, the intraclass correlation coefficient (ICC) was used with a confidence interval. ICC values below 0.5 were considered poor, between 0.70 and 0.89 reliable, and > 0.90 excellent (Landis and Koch, 1975). In the execution time, at each test and retest time, we performed a time stability test, the response speed of each subtest was measured from the Pearson (r) coefficient: *p*-values below 0.5 were considered poor; between 0.70 and 0.89 reliable; and > 0.90 excellent (Landis and Koch, 1975).

#### Validity

##### Evidence based on internal structure

To know the internal properties of the results, principal components analysis (PCA) was used. The variance of the number of correct answers in each subtest was normalized and analyzed in a correlation matrix. From there, we analyze the coordinates and contributions.

##### Evidence based on relations to other variables

To verify the existence of a correlation between the WISC and ANAM, and WAIS and ANAM instruments, Spearman’s correlation coefficients were calculated from the raw data of each subtest, with the WISC to people aged 16 years or younger, and the WAIS being applied to people aged 17 years, or older. The significance level adopted for the study was p = 0.05. The correlation between the batteries is convergent, which means that they evaluate a similar construct (here: cognitive ability) (American Educational Research Association [AERA] et al., 2014).

## Results

### Demography

Table 4 presents demographic data for the samples. The second table, on the other hand, presents the results of means and standard deviation of the Ped-ANAM divided by the same age group that performed the WISC and WAIS scale tests. Table 5 presents Ped-Anam hits by subtest.

**TABLE 4:** Sociodemographic characteristics.

Group 1 *N* = 230			Group 2 *N* = 51		Group 3 (WISC) *N* = 17		Group 3 (WAIS) *N* = 37	
			
Characteristics	N	%	N	%	N	%	N	%
**Age group**								
9–16	96	41.74	21	41.18	17	100		
≥17	134	58.26	30	58.82			37	100
**Education level**								
Elementary school finished	20	8.7	5	9.8	11	64.71		
High school unfinished	41	17.83	8	15.69	6	29.41		
High school finished	69	30	15	29.41			2	2.78
University unfinished	50	21.74	10	19.61			22	61.11
University finished	35	15.22	8	15.69			9	25
Postgraduate	15	6.52	5	9.80			4	11.11
**Sex**								
Female	150	65.22	30	58.82	11	66.67	25	67.57
Male	80	34.78	21	41.18	6	29.41	12	32.43

WISC, Wechsler Intelligence Scale for Children; WAIS, Wechsler Adult Intelligence Scale.

**TABLE 5:** Ped-ANAM hits by subtest.

	Total sample *N* = 230	Age group 1 *N* = 96	Age group 2 *N* = 134
			
Subtest	Mean (*SD*)	Mean (*SD*)	Mean (*SD*)
Procedural reaction time	19.12 (1.82)	18.68 (1.55)	19.19 (1.65)
Code substitution	29.25 (6.09)	29.66 (4.06)	28.87 (5.88)
Logical relations	19.49 (1.20)	18.91 (1.37)	19.47 (1.01)
Spatial processing	17.76 (2.04)	17.54 (2.80)	17.96 (2.10)
Mathematical processing	18.10 (2.07)	17.89 (1.98)	18.75 (1.34)
Matching grids	70.05 (6.56)	69.25 (2.32)	69.91 (1.20)
Matching to sample	19.12 (1.82)	18.68 (1.55)	19.19 (1.65)
Memory search	16.67 (3.95)	15.40 (3.92)	16.06 (3.94)
Effort	4.88 (5.93)	7.60 (4.78)	3.60 (4.06)

Age group 1 represent range of age 9–16 years. Age group 2 represent over 17 years old.

### Reliability

Internal consistency: The overall Cronbach’s α value of the Ped-ANAM battery was 0.860, suggesting good internal consistency. For individual Ped-ANAM tests, Cronbach’s α values were 0.83 for code substitution, 0.83 for logical relations, 0.84 for matching grids, 0.84 for matching to sample, 0.92 for mathematical processing, 0.88 for memory search, 0.94 for spatial processing, 0.85 for effort, 0.72 for procedural reaction time, and 0.79 for continuous memory performance, respectively (Table 6).

**TABLE 6:** Ped-ANAM battery internal consistency (Alpha Cronbach).

	Total sample *N* = 230
Subtest	Cronbach alpha
Procedural reaction time	0.72
Code substitution	0.83
Logical relations	0.82
Spatial processing	0.94
Running memory	0.79
Mathematical processing	0.92
Matching grids	0.84
Matching to sample	0.84
Memory search	0.88
Effort	0.85

Test/retest reliability: The intraclass correlation index (ICC), observed in the different subtests, was code substitution-learning 0.94, matching to sample 0.87, spatial processing 0.87, effort represented 0.80, memory search 0.76, matching grids 0.75, math processing 0.70, procedural reaction time 0.61, continuous memory performance test running 0.59, and logical relations 0.89 (Table 7).

**TABLE 7:** Intraclass correlation index of Ped-ANAM test-retest reliability.

	Total sample *N* = 51
Subtest	ICC
Procedural reaction time	0.72
Code substitution	0.83
Logical relations	0.82
Spatial processing	0.94
Running memory	0.79
Mathematical processing	0.92
Matching grids	0.84
Matching to sample	0.84
Memory search	0.88
Effort	0.85

When evaluating time stability, Pearson’s coefficients (r), for each of the Ped-ANAM subtests, was as it follows: Code Substitution-Learning *r* = 0.90, Matching Grids *r* = 0.90, Spatial Processing *r* = 0.89, Logical Relations *r* = 0.70, Running Memory Continuous Performance Test *r* = 0.64, Matching to Sample *r* = 0.62, Matching to Sample *r* = 0.62, Memory Search *r* = 0.50, Procedural Reaction Time *r* = 0.48, and Mathematical Processing *r* = –0.29 (Table 8).

**TABLE 8:** Correlation between execution speed in each subtest in test-retest evaluation (Pearson correlation).

	Total sample *N* = 51
Subtest	Pearson
Procedural reaction time	0.72
Code substitution	0.83
Logical relations	0.82
Spatial processing	0.94
Running memory	0.79
Mathematical processing	0.92
Matching grids	0.84
Matching to sample	0.84
Memory search	0.88
Effort	0.85

#### Validity

##### Evidence based on internal structure

**FIGURE 2:**
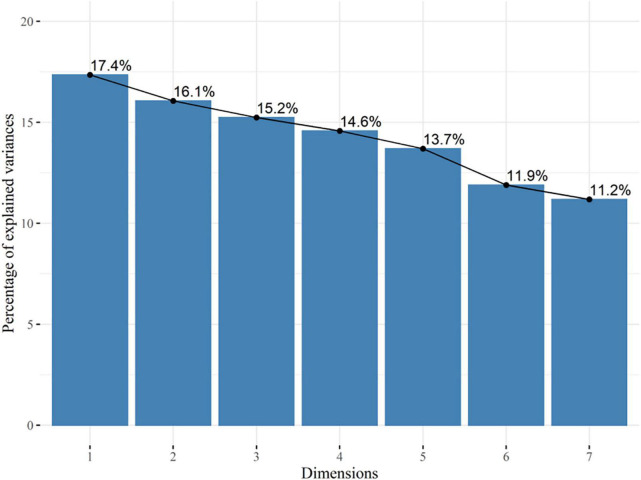
Principal component analysis (PCA) Ped-ANAM.

Figure 2 shows the percentages of variability for each principal component and Table 9 shows the coordinates.

**TABLE 9:** Coordinates of a principal components analysis in Ped-ANAM.

	Eigenvalue	Percentage of variance	Cumulative percentage of variance
Comp 1	1.21	17.35	17.35
Comp 2	1.12	16.06	33.41
Comp 3	1.07	15.24	48.65
Comp 4	1.02	14.57	63.23
Comp 5	0.96	13.69	76.92
Comp 6	0.83	11.90	88.82
Comp 7	0.78	11.18	100.00

##### Evidence based on relations to other variables

Significant associations between Ped-ANAM subtests and the WISC are summarized in Table 10. There were moderate associations between simple reaction time when compared to visual puzzles (rho = 64 *p* = 0.007); reasoning in relation to matching grids (rho = 0.63 *p* = 0.006); and visual vs. spatial puzzles (rho = 0.55 *p* = 0.02). Associations of the WAIS and Ped-ANAM are summarized in Table 11. Notably, there were moderately strong positive associations between coding, procedural reaction time (rho = 0.55; *p* = 0.02), and mathematical processing (rho = 54 *p* = 0.03), respectively, as well as digit span and simple reaction time (rho = 0.43; *p* = 0.04).

**TABLE 10:** Correlation between WISC and Ped-ANAM for children under 16 years old (*n* = 17).

WISC	Ped-ANAM (Spearman correlation)					
Figure weights	Spatial processing (rho = 0.55)	Mathematical processing (rho = 0.54)	Matching grids (rho = 0.46)	Memory search (rho = 0.51)		
Visual puzzles	Spatial processing (rho = 0.55)	Mathematical processing (rho = 0.50)	Matching grids (rho = 0.57)			
Reasoning	Spatial processing (rho = 0.48)	Matching grids (rho = 0.63)	Matching to sample (rho = 0.56)	Simplert (rho = 0.54)	Running (rho = 0.44)	Memorys (rho = 0.46)
Block design	Mathematical processing (rho = 0.43)	Matching grids (rho = 0.48)	Matching to sample (rho = 0.46)	Reaction time (rho = 0.41)	Simplert (rho = 0.52)	
Letter-number sequencing	Mathematical processing (rho = 0.40)	Matching to sample (rho = 0.50)				
Coding	Matching to sample (rho = 0.50)					
Reasoning with words	Matching to sample (rho = 0.49)					
Arithmetic	Reaction (rho = 0.54)	Mathp (rho = 0.40)	Matchings (rho = 0.45)	Simplert (rho = 0.45)		

WISC, wechsler intelligence scale for children.

**TABLE 11:** Correlation between WAIS and Ped-ANAM for people over 17 years old (*n* = 37).

WAIS	Ped-ANAM (Spearman correlation)				
Coding	Procedural reaction time (rho = 0.55)	Matching grids (rho = 0.40)	Mathematical processing (rho = 0.54)	Matching to sample (rho = 0.69)	Memory search (rho = 0.40)
Similarities	Mathematical processing (rho = 0.40)				
Matrix reasoning	Mathematical processing (rho = 0.40)	Spatial processing (rho = 0.40)			
Digit Span	Simplert (rho = 0.43)				

WAIS, wechsler adult intelligence scale.

The Ped-ANAM subtest with the highest number of associations was Mathematical Processing with eight associations. Only the Code Substitution and Logical Relations had lower correlations than expected for convergent validity (Bauer et al., 2012; American Educational Research Association [AERA] et al., 2014). Reasoning was the subtest of the gold standard scales with the highest number of associations with the Ped-ANAM, 6 in total. The 37 associations between the Ped-ANAM and the gold standard scales show us a good performance of validity in view of the constructs of both (Reeves et al., 1996; Wechsler, 1997; Reeves and Bleiberg, 2004; Zanarella, 2015).

## Discussion

Herein, we report the successful cross-cultural validation of the Brazilian-Portuguese translation of the English version of Ped-ANAM. All steps of the process of translation and cross-cultural adaptation were successfully completed (Beaton et al., 2000). The translation procedure for an instrument comprises its cultural and contextual adequacy (Beaton et al., 2000). In this sense, when adapting the tool to a new culture, it is necessary to prove the semantic, idiomatic, experiential, and conceptual equivalence of the items, as well as their psychometric properties (Hambleton, 2005). For this, some terms were not translated literally, and much attention was given to possible modifications concerning the contents of the original version, according to the cultural context of its target population since the literal correspondence of a term does not necessarily imply the same understanding for different cultures (Mattos et al., 2006). Much care was also taken to select words easier to interpret and national in scope, avoiding regionality (Mattos et al., 2006).

In addition, the final version of the instrument kept the format and the sequence of the questions identical to those in the original version. Traditionally, research with printed psychological tests presents a pilot study stage to verify the proper functioning of the instrument, followed by validity research (Hutz et al., 2015). With Ped-ANAM, the changes were implemented directly in the software and it became, therefore, a more economical and faster procedure (Hutz et al., 2015).

Our study followed the suggestions of the paper published, in a joint position, by the American Academy of Clinical Neuropsychology and the National Academy of Neuropsychology, on principles and practices for the development and use of computerized neuropsychological assessment devices (Bauer et al., 2012).

We observed that reliability presented good internal (general Cronbach’s alpha value of 0.860). Cronbach’s alpha is a statistical test, often used to estimate patterns of convergence and divergence of psychological and educational instruments (Mattos et al., 2006). The highest Cronbach’s alpha coefficients were obtained for spatial and mathematical processing.

The test-retest technique, in addition to being recommended, is consistent. Several studies have addressed the ANAM test-retest in varying samples and various retest intervals (e.g., Kaminski et al., 2009; Cole et al., 2013; Vincent et al., 2018). In this study, the ICC data ranged from 0.59 to 0.94, demonstrating good reliability (Shrout and Fleiss, 1979; Conover, 1999). Corroborating with the existing literature, the highest reliabilities were observed for tasks with the highest CI (i.e., matching to sample, code substitution), while the lowest reliabilities were observed for the simple reaction time (SRT), which is the first battery subtest and measurement that indicates reaction time, basic neural processing with an emphasis on motor activity (Shrout and Fleiss, 1979; Reeves and Bleiberg, 2004; Vincent et al., 2018). These results are consistent with other SRT assessments in more diverse samples (Vincent et al., 2018) and for other traditional processing speed tests (Strauss et al., 2006), with the lowest reliabilities for SRT, probably due to the tendency of healthy individuals to exhibit near-ceiling levels of SRT performance, thus restricting the range of performance variability (Vincent et al., 2012).

To strengthen the reliability data, we included time stability, which ranged from Code Substitution-Learning 0.90, Matching Grids 0.90 to Mathematical Processing –0.29. The raw data results remained stable and strongly correlated. Only mathematical processing had a negative result, which suggests a learning effect (Calamia et al., 2012). The stability of a measure is the degree to which similar results are obtained at two different times. It is the estimate consistency of the measurement’s repetitions (Souza et al., 2017).

Validity refers to the fact that an instrument measures exactly what it purports to measure (Souza et al., 2017). There are different ways to carry out the validity analysis on batteries. In this study, it is an initial validation with our sample. After reliability, we analyzed the internal structure using PCA the results indicate that there is probably no grouping based on the evaluated variables, which is also observed in the percentages of variability of the main components, where very discrepant percentage values are not observed between the first two main components and the other components. The conceptual framework for a test may imply a single dimension of behavior, or it may posit several components that are each expected to be homogeneous, but that are also distinct from each other. This indicates a kind of multidimensionality that may be unexpected or may conform to the test framework (American Educational Research Association [AERA] et al., 2014).

Evidence based on relations to other variables used in this study was based on the Wechsler batteries, a gold standard tool to diagnose CI (Wechsler, 1997; Zanarella, 2015). Ped-ANAM is a screening battery (Reeves et al., 1996; Reeves and Bleiberg, 2004).

As expected, we have noticed moderate association with WAIS and WISC, its established measures of cognition, supporting that Ped-ANAM measures a similar construct, or even the same). In this study, the correlation ranged from fair to excellent, with no negative correlations, which suggests convergent validity.

As this is the first translation and validation study of Ped-ANAM into Brazilian-Portuguese, we used the raw data from the test and, after, transformed it into z score when comparing Ped-ANAM to the criterion standards (WAIS, WICS). WISC and Ped-ANAM showed twenty-seven associations out of 90 possible associations (30%) from the expected validation to convergent validity. WAIS and Ped-ANAM showed eight associations (possible associations: 85, 10%).

Both WISC and WAIS have structural models that include four factors: verbal comprehension, perceptual reasoning, working memory, and processing speed. The main difference between the two instruments is that WISC includes children, aged between 6 and 16, and WAIS is for adults over 16 years of age. In the present study, WISC presented closer associations than WAIS with Ped-ANAM, which may be due to measurement methods and similar graphs. In addition, although the Ped-ANAM is available for application to older adolescents and adults, it is mainly a pediatric version instrument, with the projection performed mainly for children, which could be the reason why there is a greater approximation between the results obtained in it and WISC (Reeves et al., 1996; Brunner et al., 2007). There may also have been fluctuations in test performance, although the environment was set up to protect against such errors (Hutz et al., 2015).

The promise and potential of a computerized battery for screening CI has been widely discussed (Beaton et al., 2000; Gottschalk et al., 2000; Kabat et al., 2001; Brunner et al., 2007; Vega-Fernandez et al., 2015). One study presented the mean scores of Ped-ANAM that differed significantly among children with different levels of cognitive performance and allowed the detection of moderate or severe CI with a sensitivity of 100% and specificity of 86%, thus showing to be an excellent battery (Brunner et al., 2007). When compared with a systematic review study on the validation of screening instruments in the literature, they showed similar values (Landis and Koch, 1975; Fleiss, 1981; Streiner and Norman, 1995; Oliveira et al., 2011).

It is noteworthy that the cross-cultural adaptation strategies of research instruments are numerous, and the process described in this article was chosen based on the characteristics of the software and the study design (Nunnally, 1978; Beaton et al., 2000; Hambleton, 2005; Mattos et al., 2006; Hutz et al., 2015). Which showed us that Ped-ANAM preserves the coherence and integration of understood items and constructs (Nunnally, 1978; Beaton et al., 2000; Hambleton, 2005; Mattos et al., 2006; Hutz et al., 2015). The study showed evidence of reliability and initial validity, which makes the Ped-ANAM a reliable tool for screening cognitive function (Nunnally, 1978; Hambleton, 2005; Mattos et al., 2006; Brunner et al., 2007; Hutz et al., 2015). It is a good battery for tracking CI and exploring different areas of cognition. The translation and initial validation of the device suggests that it can be a useful screening tool of cognitive ability in individuals speaking Brazilian Portuguese (Nunnally, 1978; Beaton et al., 2000; Hambleton, 2005; Mattos et al., 2006; Hutz et al., 2015).

### Future directions

In addition to the practical application of Ped-ANAM, its use in future studies will allow better comparison with multicenter data already existing in the scientific community and may also help clinical professionals and researchers in their analysis, thus favoring technical improvement, cost reduction, and improvement in the quality of services provided. Due to the lack of instruments validated for clinical use with these characteristics in Brazil, this is the first translation and cross-cultural adaptation of Ped-ANAM. Future research should continue to examine the psychometric properties of Ped-ANAM, particularly across multiple time intervals and across more diverse samples, as well as directly comparing scores obtained when administered.

### Limitations

Regarding the limitations of this study, one possibility is the cross-cultural validation of another language. There are difficulties and limitations inherent to the process of adapting instruments from other countries, mainly due to cultural and language differences. Our study carried out the translation and validation based on the literature guidelines; however, there were semantic or conceptual differences between the original instrument and the Brazilian version achieved, precisely due to the items that did not correspond to the Brazilian cultural reality (Nunnally, 1978; Beaton et al., 2000; Hambleton, 2005; Mattos et al., 2006; Hutz et al., 2015).

## Conclusion

In conclusion, the present study provides initial psychometric data for the Brazilian-Portuguese version of the Ped-ANAM, suggesting that its translated version has similar measurement properties, specifically feasibility, reliability and validity.

## Data availability statement

The raw data supporting the conclusions of this article will be made available by the authors, without undue reservation.

## Ethics statement

The studies involving human participants were reviewed and approved by the University of Campinas (CAAE 39750914.3.1001.5404). Written informed consent to participate in this study was provided by the participants’ legal guardian/next of kin.

## Author contributions

JA conceived and designed the analysis, collected the data, contributed to data and analysis tools, performed the analysis, wrote the manuscript, reviewed, and approved final manuscript. SK collected the data, contributed to data and analysis tools, wrote the manuscript, reviewed, and approved final manuscript. CE, CS, and CS-M contributed to data and analysis tools, reviewed, and approved final manuscript. FC collected the data, reviewed, and approved final manuscript. RM collected the data, wrote the manuscript, reviewed, and approved final manuscript. PF and HB conceived and designed the analysis, wrote the manuscript, contributed data or analysis tools, reviewed, and approved final manuscript. SA conceived and designed the analysis, collected the data, contributed to data and analysis tools, performed the analysis, wrote the manuscript, and approved final manuscript. All authors contributed to the article and approved the submitted version.
