# Supplementary material for: Cross-cultural adaptation and initial validation of the Brazilian-Portuguese version of the pediatric automated neuropsychological assessment metrics
Source: Front Psychol. 2022 Sep 16;13:945425. doi: 10.3389/fpsyg.2022.945425 (PMC9524254; doi:10.3389/fpsyg.2022.945425)
Supplement: Supplementary file 1 [file Table_1.docx]

**Supplementary file 1.** Modifications made in the translation stage.

| **Original item** | **Translation** | **Adaptation** |
| --- | --- | --- |
| A house has windows | Uma casa tem janelas | A casa tem janelas |
| Hats go on your head | Chapéus vão na sua cabeça | O chapéu vai na sua cabeça |
| Hats go on your feet | Chapéus vão em seus pés | O chapéu vai nos seus pés |
| Shoes go on your feet | Sapatos vão nos seus pés | O sapato vai nos seus pés |
| Shoes go on your head | Sapatos vão na sua cabeça | O sapato vai na sua cabeça |
